# Sulfur(VI) Fluoride Exchange Chemistry in Polymer Functionalization: Post-Polymerization Modification, Interface Engineering, and Biopolymer Conjugation

**DOI:** 10.3390/molecules31162832

**Published:** 2026-08-13

**Authors:** Xiaohe Zhang, Pengrui Du, Lingxia Chen, Minlong Wang, Xiangyu Liu, Ruoyan Yang, Jie An

**Affiliations:** 1 Department of Nutrition and Health, China Agricultural University, Beijing 100193, China; b20223311102@cau.edu.cn (X.Z.); b20253311591@cau.edu.cn (P.D.); mlwang@cau.edu.cn (M.W.); jie_an@cau.edu.cn (J.A.); 2 Faculty of Science and Engineering, University of Nottingham Ningbo China, Ningbo 315100, China

**Keywords:** sulfur(VI) fluoride exchange, post-polymerization modification, surface chemistry, biopolymer conjugation, covalent capture

## Abstract

Sulfur(VI) fluoride exchange (SuFEx) chemistry is a powerful click reaction for modular synthesis, distinguished by high chemoselectivity, broad functional-group tolerance and the formation of robust sulfur(VI)-based linkages. These attributes are particularly valuable for polymer functionalization, as S(VI)–F handles on either the polymer or the modifier enable covalent coupling under controlled conditions. This review spans SuFEx-mediated post-polymerization modification and architectural control of synthetic polymers, surface, interfacial and porous-material functionalization, and SuFEx-based conjugation and covalent capture in natural and sequence-defined biopolymers. Across these contexts, we compare the advantages, supporting mechanistic and analytical evidence, current limitations and future opportunities of SuFEx-enabled polymer functionalization.

## 1. Introduction

Click chemistry was introduced by Kolb, Finn and Sharpless in 2001 as a conceptual framework for selecting reactions that enable the rapid and reliable assembly of molecular complexity from modular building blocks [1]. Rather than defining a single reaction class, the term describes transformations that are high yielding, broad in scope, selective, tolerant of common functional groups, operationally simple, and able to proceed with minimal byproducts and straightforward purification. These criteria have had particular influence in polymer science [2], where efficient coupling reactions are required not only to build new structures but also to modify preformed macromolecules, interfaces, particles and biomolecular scaffolds. In this setting, however, the standards for a “click” reaction become more demanding than in small-molecule synthesis [3,4,5]. The substrate is often not a single purifiable compound, but a distribution of polymer chains, a brush layer, a nanoparticle surface, a membrane, a pore network, or a folded biomacromolecule. Incomplete conversion, residual precursor chains, backbone degradation, uncontrolled cross-linking, shifts in number-average molecular weight and dispersity, nonuniform surface coverage, or nonspecific biological reactivity can all be obscured if success is judged only by the formation of a new covalent bond. Thus, in polymer functionalization, the central question is not simply whether a linkage can be formed, but whether the transformation is chemoselective, quantifiable, compatible with the macromolecular scaffold, and causally connected to the resulting material or biological function.

**Figure 1:**
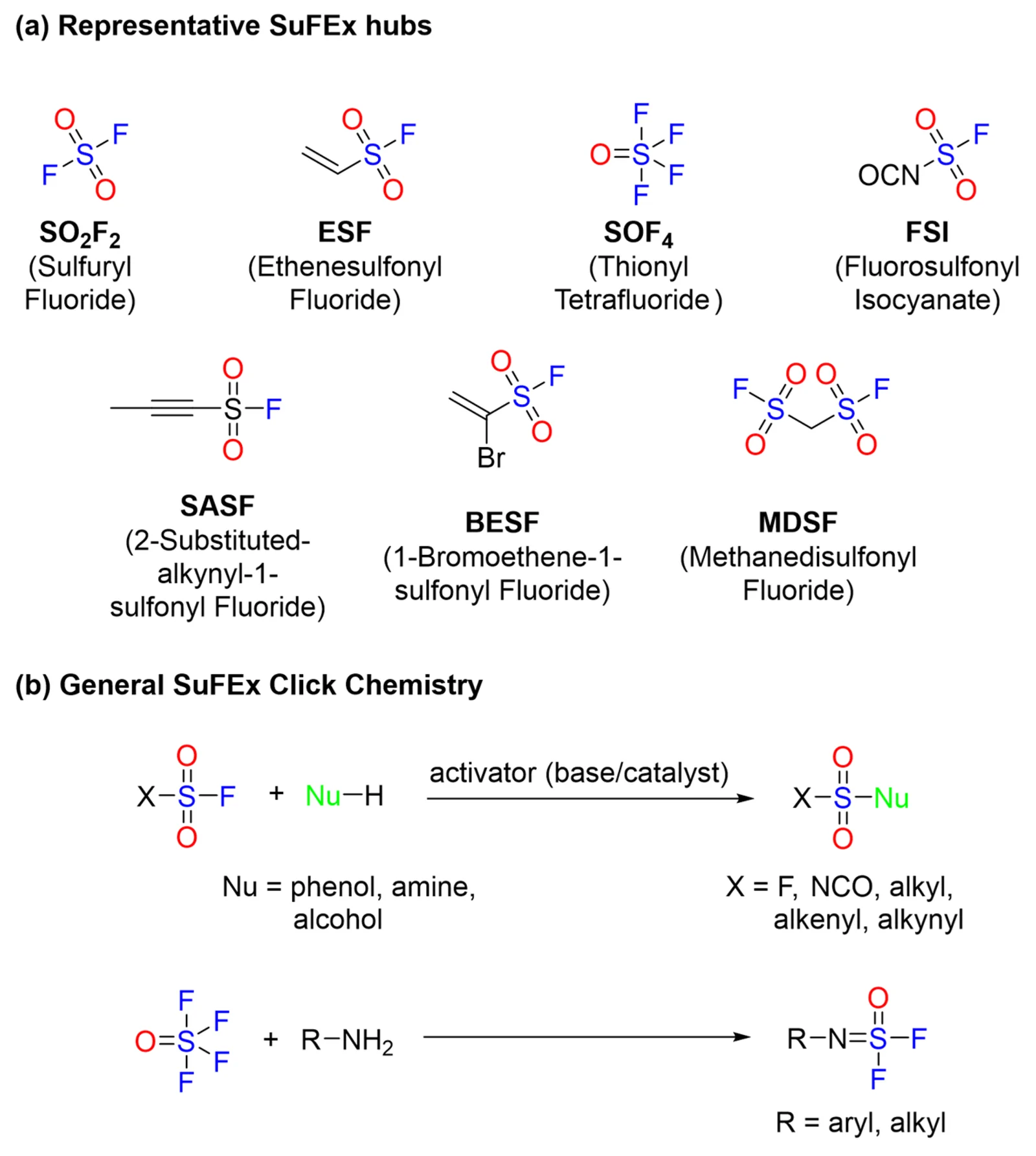
Representative SuFEx hubs and general sulfur(VI) fluoride exchange chemistry. (**a**) Representative S(VI)–F-containing hubs commonly used in SuFEx chemistry. (**b**) General SuFEx exchange of S(VI)–F handles with oxygen- or nitrogen-centered nucleophiles.

Sulfur(VI) fluoride exchange (SuFEx) chemistry provides a potentially useful approach to this problem. Introduced by Dong, Sharpless and co-workers as “another good reaction for click chemistry”, SuFEx exploits the unusual stability–reactivity balance of sulfur(VI)–fluoride bonds [6]. The reactivity of SuFEx chemistry arises from the unique stability yet controllable exchangeability of the S(VI)–F bond. Mechanistically, SuFEx proceeds through nucleophilic attack at the electrophilic sulfur(VI) center, followed by displacement of fluoride through an addition–elimination or concerted substitution pathway [6]. Sulfonyl fluorides, fluorosulfates, sulfamoyl fluorides and related S(VI)–F hubs are often sufficiently persistent to survive synthesis, purification, polymerization or materials processing, yet can undergo exchange with appropriate oxygen-, nitrogen- or other nucleophilic partners under controlled activation conditions (Figure 1a,b). Subsequent reviews and primers have established SuFEx as a versatile platform spanning organic synthesis, materials chemistry, chemical biology and polymer science [7,8]. For polymer functionalization, its value is not limited to installing sulfur(VI)-centered linkages on a polymer backbone or side chain [9,10]. Rather, SuFEx enables complementary designs in which either the macromolecular scaffold or the incoming modifier carries the S(VI)–F handle [10,11,12]. Polymer-bound sulfonyl fluoride or fluorosulfate groups can be coupled with small-molecule, macromolecular or biomolecular nucleophiles; conversely, nucleophile-bearing polymers, surfaces and biopolymers can be modified with S(VI)–F-containing molecules or ligands. This dual polarity broadens SuFEx from a handle-diversification reaction into a general strategy for post-formation covalent connection, interfacial engineering, bioconjugation and proximity-enabled covalent capture.

The breadth of SuFEx chemistry also requires careful qualification. Different S(VI)–F hubs differ substantially in electrophilicity, hydrolytic stability, steric accessibility, leaving-group behavior and compatibility with catalysts, solvents and biological media [9,11,13,14]. These differences are amplified in macromolecular environments, where chain conformation, swelling, phase separation, interfacial confinement, pore diffusion and local molecular recognition can determine whether an apparently robust small-molecule reaction remains effective. Consequently, claims of click-like performance in polymer functionalization should be linked to a defined handle, scaffold, medium and evidence set. For soluble polymers, persuasive evidence includes high conversion, retention of molecular-weight distribution, low residual precursor content and preservation of architecture [2,3,4,5]. For surfaces, brushes, membranes and porous materials, chemical conversion must be interpreted together with coverage, accessibility, diffusion and morphology retention [10,12,15]. For natural and sequence-defined biopolymers, site selectivity often arises from binding, proximity and reactive geometry, rather than from freely diffusing chemoselectivity alone [11,14]. These considerations motivate an evidence-based comparison throughout this review: SuFEx functionalization is assessed not only by bond formation, but also by how convincingly covalent modification, structural integrity and functional outcome are connected.

**Figure 2:**
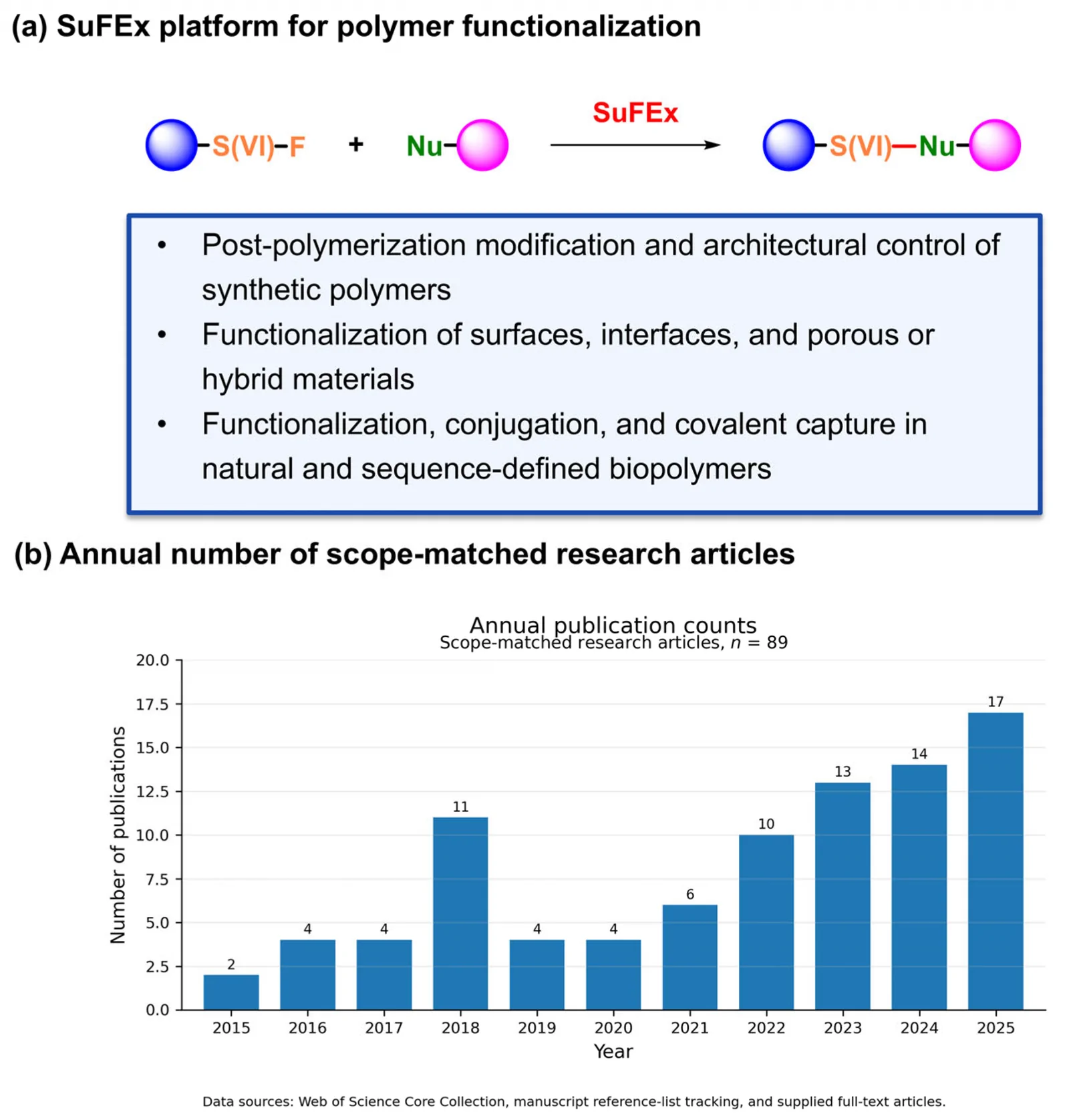
Conceptual scope and publication activity of SuFEx-enabled polymer functionalization. (**a**) Schematic coupling of an S(VI)–F-bearing component with a nucleophile-bearing partner through SuFEx, highlighting the three domains covered in this review: post-polymerization modification and architectural control of synthetic polymers; functionalization of surfaces, interfaces, and porous or hybrid materials; and functionalization, conjugation, and covalent capture in natural and sequence-defined biopolymers. (**b**) Annual number of scope-matched research articles published from 2015 to 2025 (*n* = 89). Data sources: Web of Science Core Collection, manuscript reference-list tracking, and supplied full-text articles.

Several excellent reviews have defined the foundations of this field. General SuFEx reviews and methods primers describe the reaction family and its expanding applications [7,8]; linker-focused and decade-perspective articles clarify the growing diversity of SuFEx hubs [9,13]; polymer functionalization reviews and related works establish the standards by which macromolecular transformations should be evaluated [2,3,4,5]; and surface, biofunctional polymer and chemical-biology reviews frame the constraints of interfaces and biomolecular systems [11,12,14,15]. However, SuFEx applications in soluble polymers, confined interfaces, porous materials and biopolymers are still often discussed within separate application areas, rather than compared by common criteria for post-formation covalent modification. This review addresses that gap by focusing on SuFEx as a polymer functionalization strategy across three related research fields. These three fields are condensed into a common SuFEx design, in which an S(VI)–F-bearing component is coupled with a nucleophile-bearing partner to enable post-polymerization modification and architectural control of synthetic polymers, functionalization of surfaces, interfaces and porous or hybrid materials, and conjugation or covalent capture in natural and sequence-defined biopolymers (Figure 2a). Small-molecule SuFEx methodology, general covalent drug discovery and main-chain SuFEx polymerization are outside the primary scope, except where they directly inform the design of SuFExable polymer scaffolds or leave residual S(VI)–F functionality for subsequent modification [10].

This narrative review was supported by a structured search of the Web of Science Core Collection conducted in July 2026 for records published from January 2014 to the search date. Topic searches combined “SuFEx” and variants of “sulfur(VI) fluoride exchange” with terms covering synthetic-polymer post-functionalization, surfaces/interfaces and porous materials, and natural or sequence-defined biopolymers. Studies were included when S(VI)-F chemistry was used for post-formation polymer modification, macromolecular coupling, surface or porous-material functionalization, or biopolymer conjugation/covalent capture. Small-molecule reactions, general covalent drug studies, and main-chain SuFEx polymerizations without subsequent functionalization or retained addressable S(VI)-F groups were excluded from the primary scope. Reference-list and cited-reference tracking were used to identify additional relevant reports. Within this defined scope, 89 research articles were included for 2015–2025; annual publication activity fluctuated but was concentrated in 2023–2025, while the application landscape diversified—most prominently toward biopolymer systems—and explicit process- and industrial-translation evidence remains limited (Figure 2b).

## 2. SuFEx-Mediated Post-Polymerization Functionalization of Synthetic Polymers

SuFEx-mediated functionalization of synthetic polymers is discussed here as a post-formation strategy that converts pre-existing macromolecular scaffolds into chemically and functionally differentiated materials. The section is organized according to the position of the reactive site. Pendant and side-chain functionalization primarily changes composition, polarity, charge, recognition motifs, or catalytic groups while retaining the parent chain architecture. Chain-end functionalization, polymer–polymer coupling and cyclic architecture formation instead use SuFEx to alter the identity, connectivity, or topology of entire macromolecules. Across these regimes, the central analytical requirements are the same: the reactive pair must be chemically accessible, conversion must be assigned with polymer-appropriate methods, molecular-weight distribution and backbone integrity should be retained, and the observed function should be interpreted in relation to both covalent structure and polymer morphology.

### 2.1. Pendant and Side-Chain Functionalization

Pendant and side-chain post-polymerization modification is a central route to functional polymer libraries because a single precursor can be diversified after polymerization. This strategy avoids repeated monomer synthesis and permits the installation of groups that may be incompatible with polymerization conditions. Established PPM reactions have made this approach broadly useful, yet polymer substrates impose constraints that are less pronounced in small-molecule synthesis: dense side-chain substitution can restrict diffusion and accessibility, incomplete conversion generates compositional heterogeneity, and side reactions may broaden the molecular-weight distribution or induce gelation [2,3]. SuFEx is suited to this problem because S(VI)–F motifs can be introduced as latent electrophilic handles or used as external modifiers for nucleophile-bearing polymers. In both designs, the effectiveness of the transformation depends on matching handle stability, activation mode and side-chain environment to the macromolecular scaffold.

**Figure 3:**
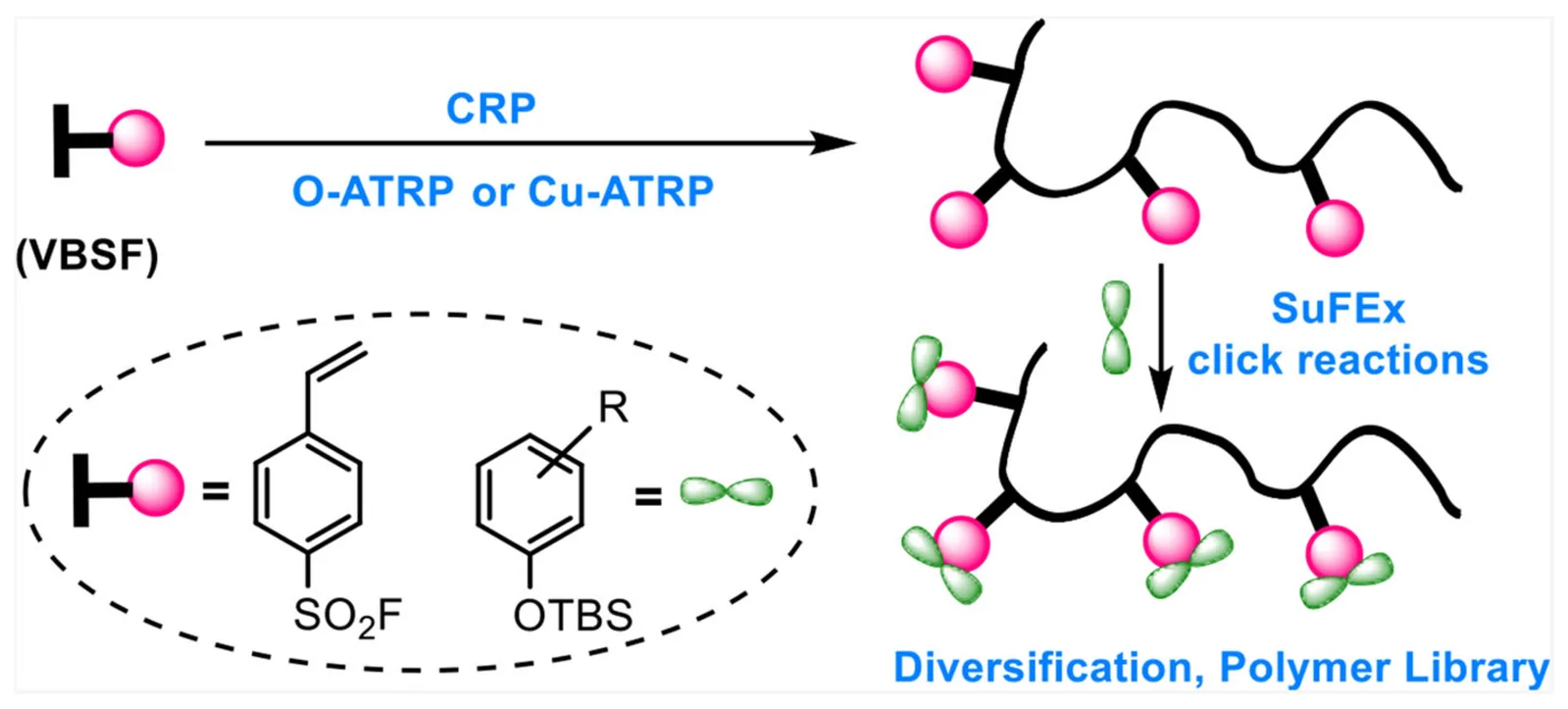
VBSF monomer-encoded sulfonyl fluoride handle strategy and subsequent SuFEx-mediated pendant diversification of poly(VBSF)-based polymers.

**Figure 4:**
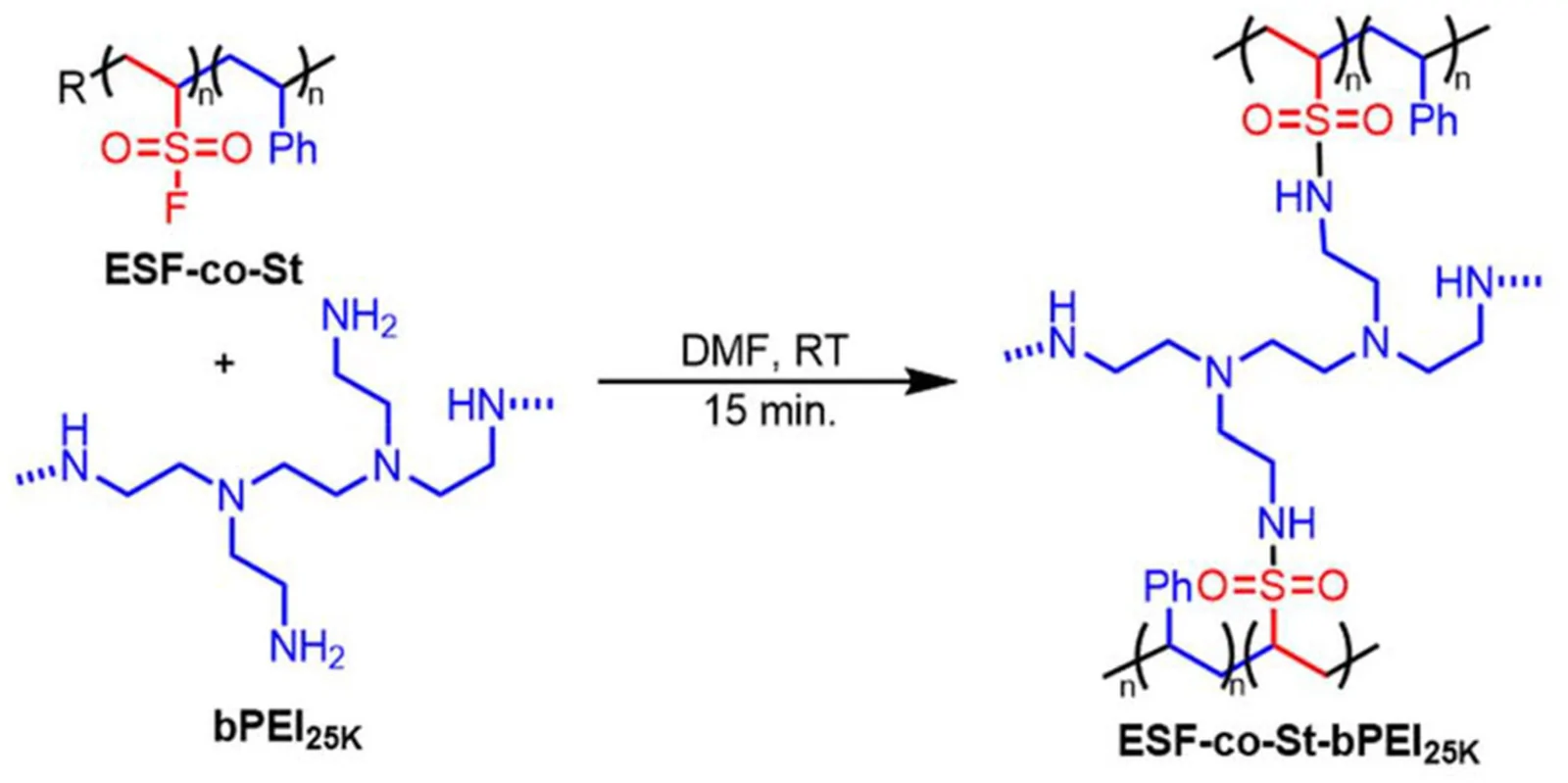
SuFEx-mediated cross-linking of ESF-co-St copolymers with branched polyethylenimine (bPEI_25k_), leading to three-dimensional gel formation.

Monomer-encoded S(VI)–F handles provide the most direct implementation of pendant SuFEx PPM. In aliphatic polycarbonates, a sulfonyl fluoride-bearing cyclic carbonate monomer was incorporated by organocatalytic ring-opening polymerization to give a pFMC precursor that could be diversified after polymerization [16]. This platform illustrates a useful design principle for degradable and biomaterial-oriented polymers: the backbone can be constructed under conditions that preserve a latent SuFEx handle, and functional groups can then be introduced at a late stage to tune self-assembly, hydrogel formation, antibacterial activity, or membrane behavior. Vinyl monomer platforms extend this logic to carbon-chain polymers. Polymerization of 4-vinylbenzenesulfonyl fluoride affords aryl sulfonyl fluoride-containing polymers that can be converted into side-chain-functionalized libraries, with ^19^F NMR and GPC supporting handle retention and post-polymerization conversion (Figure 3) [17]. Ethenesulfonyl fluoride–styrene copolymerization further shows that SuFExable sites can be incorporated through a highly activated vinyl sulfonyl fluoride monomer; the reported reactivity ratios indicate a pronounced alternating tendency, so the resulting sequence distribution should be described as feed- and conversion-dependent rather than sequence-controlled. Importantly, the resulting copolymers underwent rapid SuFEx-mediated cross-linking with branched polyethylenimine under the reported conditions, affording rigid three-dimensional gels (Figure 4) [18]. These studies establish monomer design as an efficient way to encode SuFEx reactivity, while also showing that feed composition, steric accessibility and solvent-dependent conversion remain important variables in polymer libraries.

**Figure 5:**
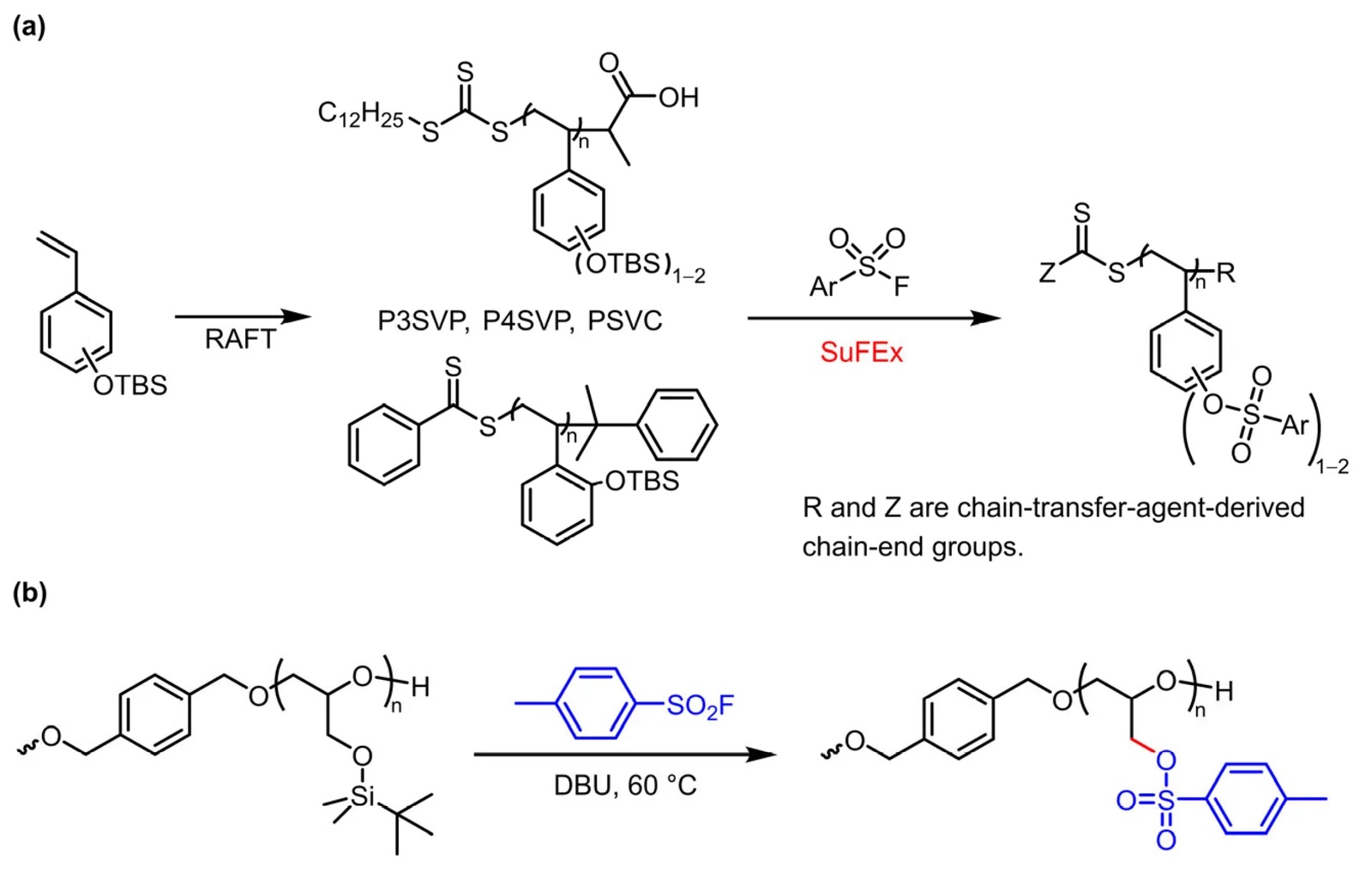
SuFEx-mediated nucleophilic side-chain modification of (**a**) PSVP and PSVC, and (**b**) silyl glycidyl ether-derived polyethers.

A complementary class of pendant modifications reverses the usual polarity of the reaction. Here the polymer displays phenol, catechol, alcohol, amine, silanol, or related nucleophilic functionality, while the incoming modifier carries the S(VI)–F hub. This approach is attractive because many synthetic polymers already contain, or can be readily converted to contain, nucleophilic side chains. Renewable phenylpropanoid-derived poly(vinylphenol) and poly(vinylcatechol) systems provide a particularly informative example (Figure 5a) [19]. Their SuFEx modification depends on substitution pattern, catechol geometry, activation mode and the electronic character of the sulfonyl fluoride partner. High conversion can be achieved in favorable combinations, whereas other conditions produce broad molecular-weight distributions or high-molecular-weight byproducts. Silyl glycidyl ether-derived polyethers offer another reverse-polarity platform, using oxygen-rich side chains generated by organocatalytic polymerization as partners for SuFEx modification (Figure 5b) [20]. These examples broaden the conceptual scope of polymer SuFEx beyond electrophilic polymer handles and emphasize activation control as a defining feature of successful reverse-polarity PPM.

**Figure 6:**
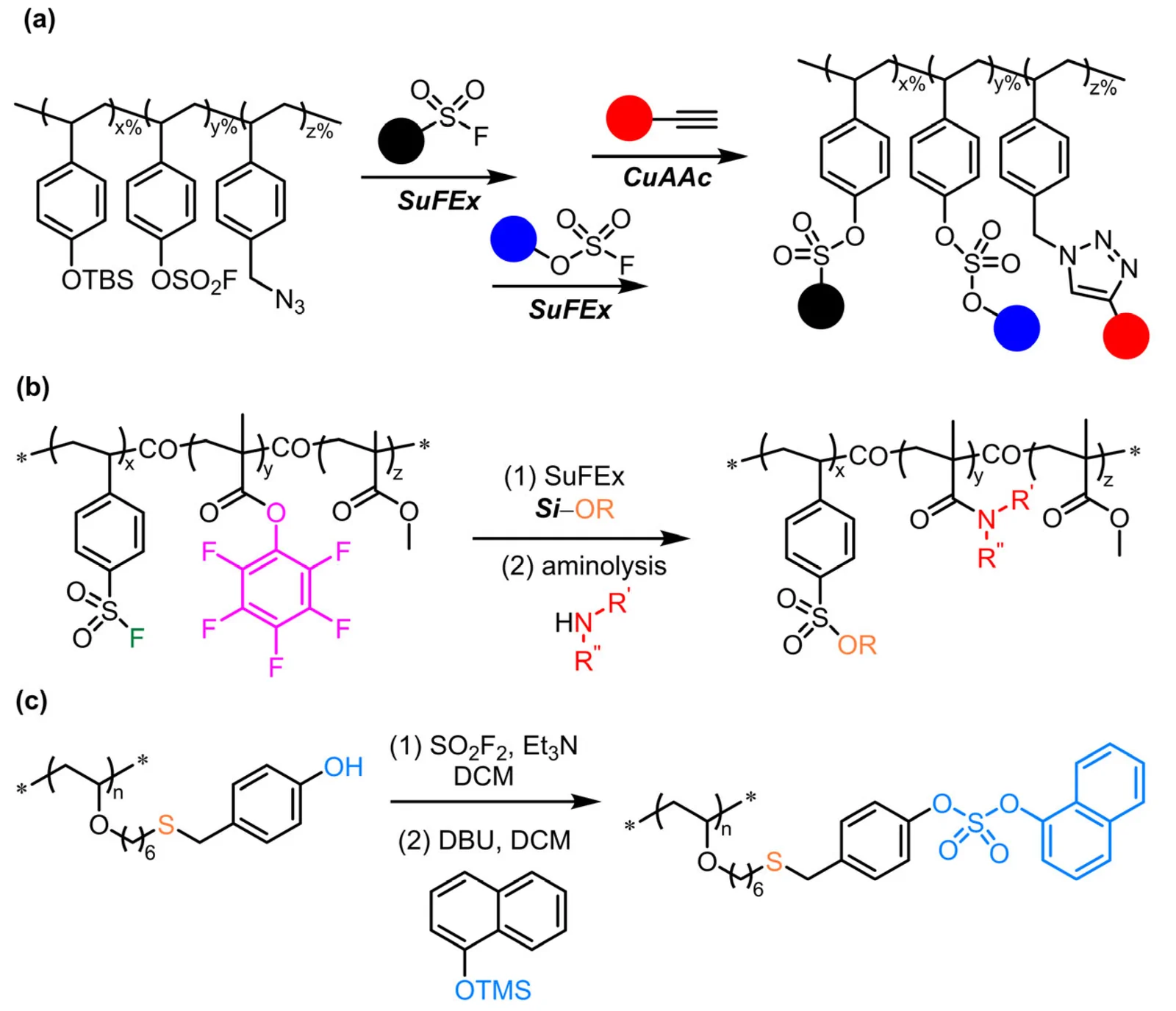
Orthogonal SuFEx platforms for sequential polymer functionalization. (**a**) Selective and orthogonal post-polymerization modification via SuFEx and CuAAC. (**b**) SuFEx-mediated selective post-polymerization modification of VBSF-based binary and ternary copolymers. (**c**) Synthesis of sulfate-functionalized isotactic polymers through sequential SuFEx modification. The asterisks (*) in panels (**b**,**c**) denote unspecified polymer chain-end groups.

Orthogonal and multifunctional platforms move pendant SuFEx from single-handle diversification toward programmed reaction sequences. The combination of SuFEx and CuAAC demonstrated that azide and S(VI)–F handles can be addressed sequentially on the same polymer, enabling covalent dye attachment and dual functionalization with support from NMR, GPC and UV–vis analysis (Figure 6a) [21]. VBSF/active-ester copolymers and stereoregular alkenyl vinyl ether polymers further show that SuFEx can be combined with amine coupling or thiol-ene chemistry to generate binary and ternary functional polymers (Figure 6b,c) [22,23]. These studies are best viewed as demonstrations of reaction orchestration: the value of the platform lies not only in the individual SuFEx step, but in the ability to preserve distinct handles until each is selectively activated. Related backbone-diverse examples, including sulfonyl fluoride-decorated polynorbornenes prepared by ROMP, show that pendant SuFEx is compatible with non-styrenic architectures, although conversion and molecular-weight retention remain substrate dependent [24].

**Figure 7:**
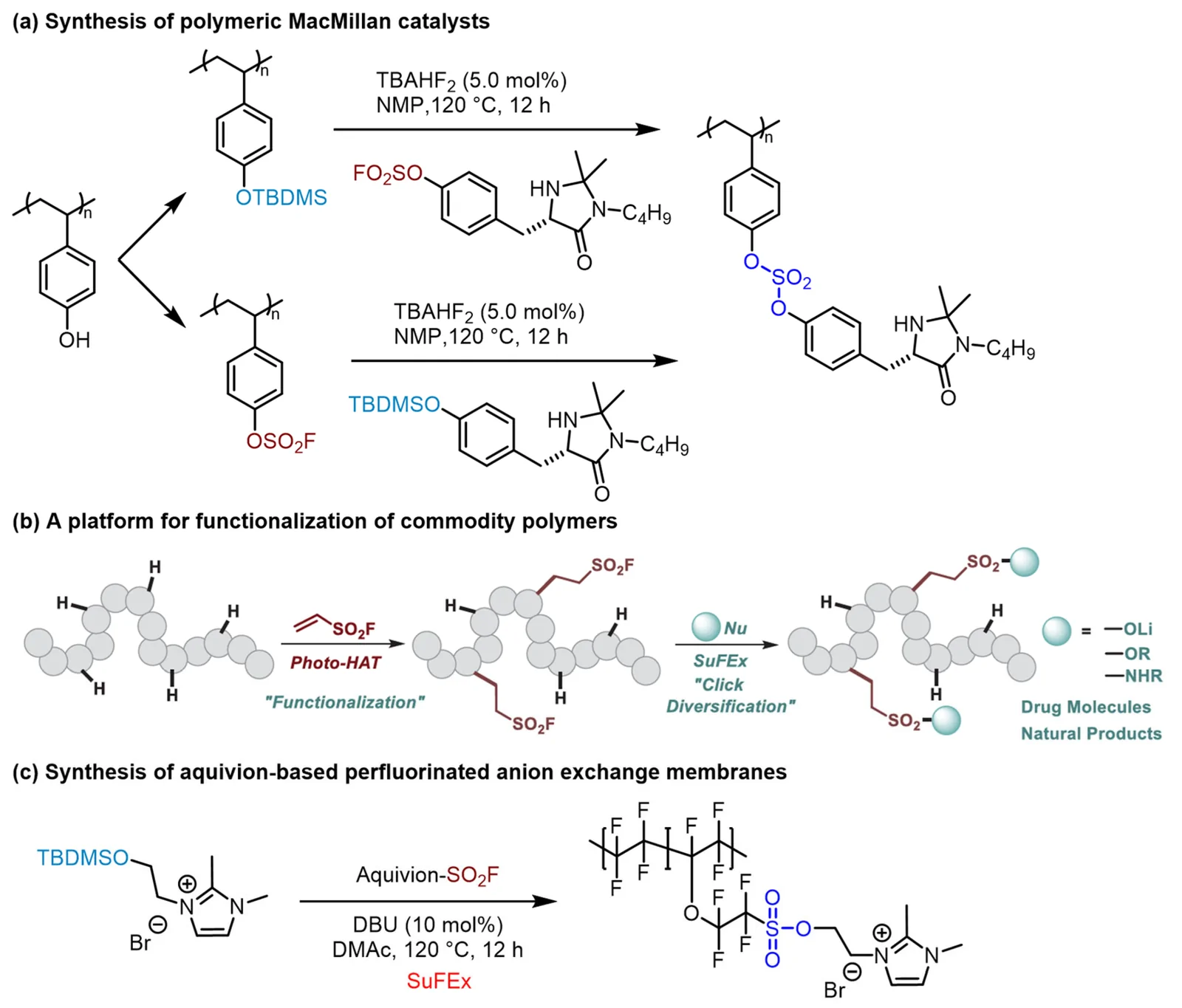
Application-oriented material design and post-polymerization modification via SuFEx chemistry. (**a**) Polymer-bound MacMillan catalysts via polysulfate scaffolds. (**b**) Photo-HAT-mediated functionalization of commodity polymers. (**c**) Aquivion-derived anion-exchange membranes.

Application-oriented examples place the covalent transformation in a broader materials context. Soluble polymer-bound MacMillan catalysts prepared through SuFEx-derived polysulfate scaffolds link side-chain grafting to asymmetric catalytic performance (Figure 7a) [25]. Photo-HAT installation of SO_2_F handles on PEG, PPO, PTHF, PCL, PE, PP and LDPE is especially notable because it transforms commodity polymers into SuFExable substrates without redesigning the monomer feed (Figure 7b) [26]. In these systems, handle loading, site distribution and possible chain damage become part of the functionalization claim. Aquivion-derived anion-exchange membranes illustrate a similar principle in ion-conducting materials: SuFEx contributes to tether construction, whereas conductivity, swelling, alkaline stability and device performance reflect the combined effects of linkage type, spacer structure, cation identity, ion-exchange capacity, phase separation and processing (Figure 7c) [27]. SOF_4_-derived helical SuFExable polymers occupy the boundary between SuFEx polymerization and PPM, because main-chain construction and residual post-formation S–F addressability are deliberately coupled [28].

Taken together, pendant and side-chain SuFEx PPM has evolved from proof-of-concept handle exchange into a flexible strategy for polymer library construction, orthogonal functionalization and materials translation. Monomer-encoded S(VI)–F polymers provide direct access to latent electrophilic scaffolds; reverse-polarity systems exploit nucleophilic polymer side chains; orthogonal platforms integrate SuFEx with other click reactions; and commodity-polymer strategies extend the method to pre-existing materials. The most persuasive studies connect handle placement, conversion, molecular-weight retention and function in a single evidence chain. This standard is essential because the properties of functional polymers are rarely determined by covalent composition alone, but by the interplay of linkage chemistry, architecture, morphology and processing history.

### 2.2. Chain-End Functionalization, Polymer–Polymer Coupling and Cyclic Architectures

Chain-end functionalization and architectural control represent a more stringent use of SuFEx chemistry. In side-chain PPM, incomplete conversion leaves unmodified repeat units within a chain distribution; in chain-end chemistry, incomplete conversion leaves entire precursor chains that can be difficult to separate from the desired product. This distinction becomes even more important in polymer–polymer coupling and cyclic polymer synthesis, where the target structure is defined by macromolecular connectivity rather than by the presence of a single new bond. SuFEx is attractive in this setting because S(VI)–F handles, aryl silyl ethers and related complementary groups can be incorporated at chain termini and activated under conditions compatible with common controlled-polymerization products. The principal challenge is analytical rather than conceptual: terminal conversion, residual precursor content, dispersity and topology must be evaluated at the level of the polymer population.

**Figure 8:**
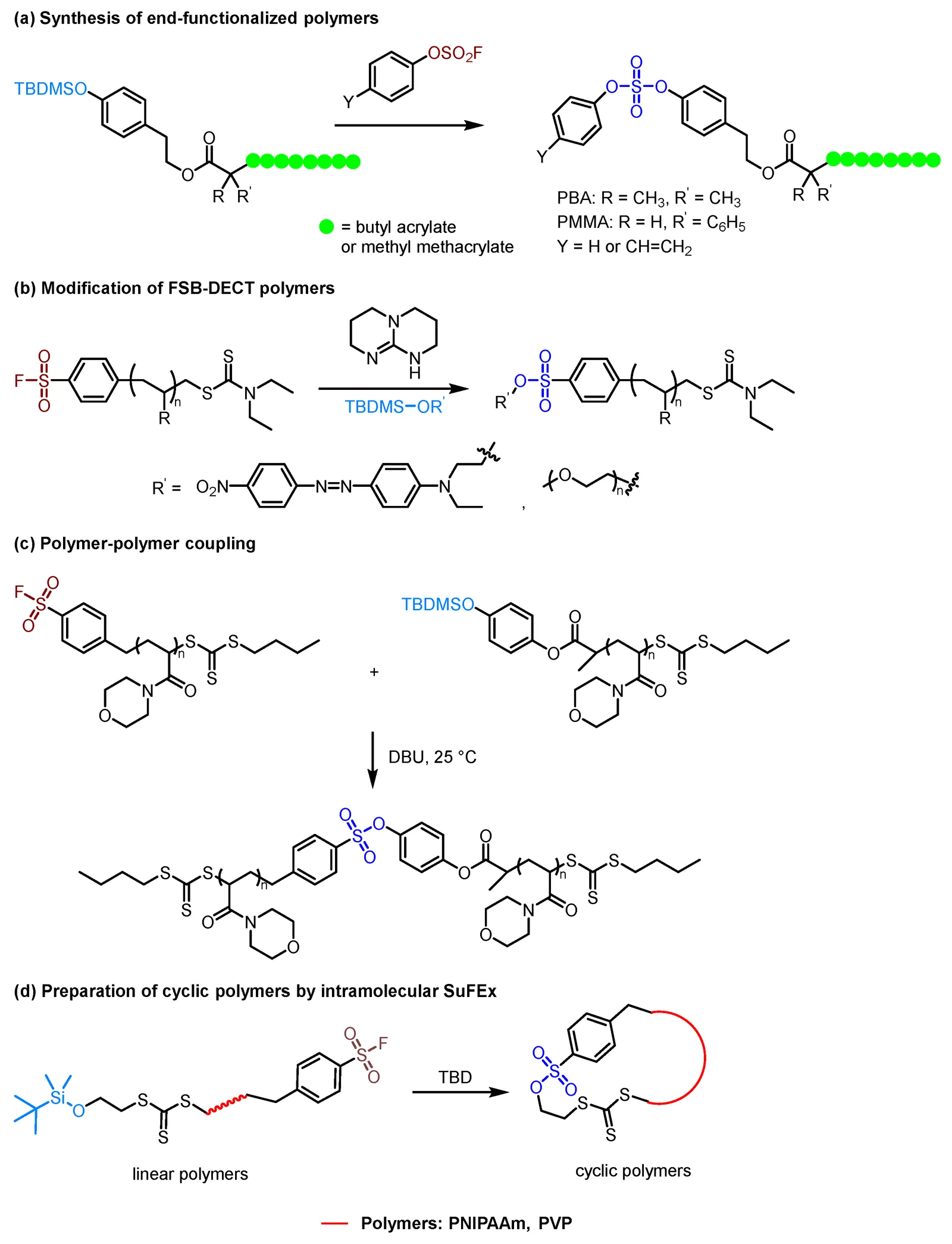
Post-polymerization chain-end modification and architecture construction via SuFEx. (**a**) SuFEx modification of AGET-ATRP-derived TBDMS end-functionalized polymers. (**b**) SuFEx modification of sulfonyl fluoride-terminated photoiniferter polymers. (**c**) SuFEx-mediated polymer–polymer coupling of complementary RAFT polymers. (**d**) Intramolecular SuFEx cyclization to cyclic polymers.

Early chain-end studies established the feasibility of SuFEx as a terminal diversification reaction. AGET ATRP provided polymers bearing aryl tert-butyldimethylsilyl ether end groups that reacted with aryl fluorosulfate partners, with ^1^H NMR end-group integration and GPC/UV dye detection supporting efficient chain-end modification (Figure 8a) [29]. Photoiniferter-derived sulfonyl fluoride-terminated polymers, including PNIPAAm, PGMA and PVAc, were likewise modified through SuFEx chemistry, and GPC shifts supported formation of PEG-containing block structures (Figure 8b) [30]. These examples introduced SuFEx into controlled-polymer platforms and demonstrated its compatibility with late-stage terminal modification. At the same time, the use of excess small-molecule reagents, residual low-molecular-weight species and dispersity changes illustrate why end-group conversion should not be equated automatically with complete chain-population conversion. Polymer–polymer coupling further raises this requirement. The equimolar coupling of complementary RAFT polymers reported by Brendel and co-workers remains an important benchmark because SEC deconvolution, catalyst screening and solvent/medium comparisons were used to distinguish efficient ligation from less favorable polymer/catalyst combinations (Figure 8c) [31]. Within the same equimolar pNAM–SO_2_F/pNAM–O–TBDMS pair in DMF at room temperature, DBU, TBAF, and MTBD at 0.30 equiv gave SEC-deconvoluted coupling efficiencies over 92% in 2 h [31]. The study shows that SuFEx can function as a rapid macromolecular ligation reaction, while retaining a clear dependence on polymer identity, medium and activation mode.

SuFEx also provides access to differentiated termini and cyclic topologies. α,ω-Heterobifunctional PVP prepared with a bis-clickable RAFT reagent carries azide and sulfonyl fluoride termini, enabling orthogonal terminal postreactions, exemplified by SuFEx-based PEG coupling and CuAAC-based dye labeling [32]. The platform demonstrates the utility of SuFEx in dual-end design, although dispersity and residual precursor quantification remain important for precise architectural claims. Intramolecular SuFEx ring closure offers a more topology-specific application (Figure 8d) [33]. In this case, high-dilution conditions, disappearance of the ^19^F signal, end-group NMR changes, SEC hydrodynamic-volume contraction and MALDI-TOF analysis collectively support cyclic polymer formation. The example illustrates the appropriate evidentiary standard for topological assignment: no single measurement is decisive, but convergent spectroscopic, chromatographic and mass-spectrometric evidence can establish the transformation with substantially greater confidence. Chain-end and architecture-directed SuFEx chemistry has progressed from terminal proof-of-reactivity to macromolecular ligation and topology control, with future advances depending on more quantitative accounting of residual precursors and product populations.

## 3. SuFEx-Enabled Surface, Interfacial and Porous-Material Functionalization

SuFEx chemistry acquires a different character when it is transferred from soluble polymer chains to surfaces, interfaces and porous materials. In these environments, the reactive site is immobilized within a brush, coating, film, pore wall, particle, carbon surface, or inorganic framework, and the observed outcome reflects both covalent exchange and spatial organization. Reagent diffusion, swelling, grafting density, surface curvature, pore connectivity and framework stability can all become as important as intrinsic S(VI)–F reactivity. SuFEx-enabled interface engineering is therefore best discussed as a sequence of increasingly confined reaction environments, rather than as a direct extension of solution-phase PPM.

This section follows that progression. Organic polymer brushes and coatings provide the most chemically transparent examples of surface-confined SuFEx PPM. Multilayer films, responsive membranes and particles then introduce assembly, transport and internal accessibility as additional design variables. Carbon, MOF and inorganic interfaces broaden the discussion toward polymer-relevant composite surfaces and hybrid porous materials. Across these systems, SuFEx is most valuable when it offers late-stage installation of a functional group while preserving the underlying surface architecture. The principal limitation is not the absence of reactivity, but the difficulty of assigning where the reaction occurs and how directly it controls the measured surface or transport property.

### 3.1. Organic Polymer Surfaces, Brushes, Films and Nanochannels

**Figure 9:**
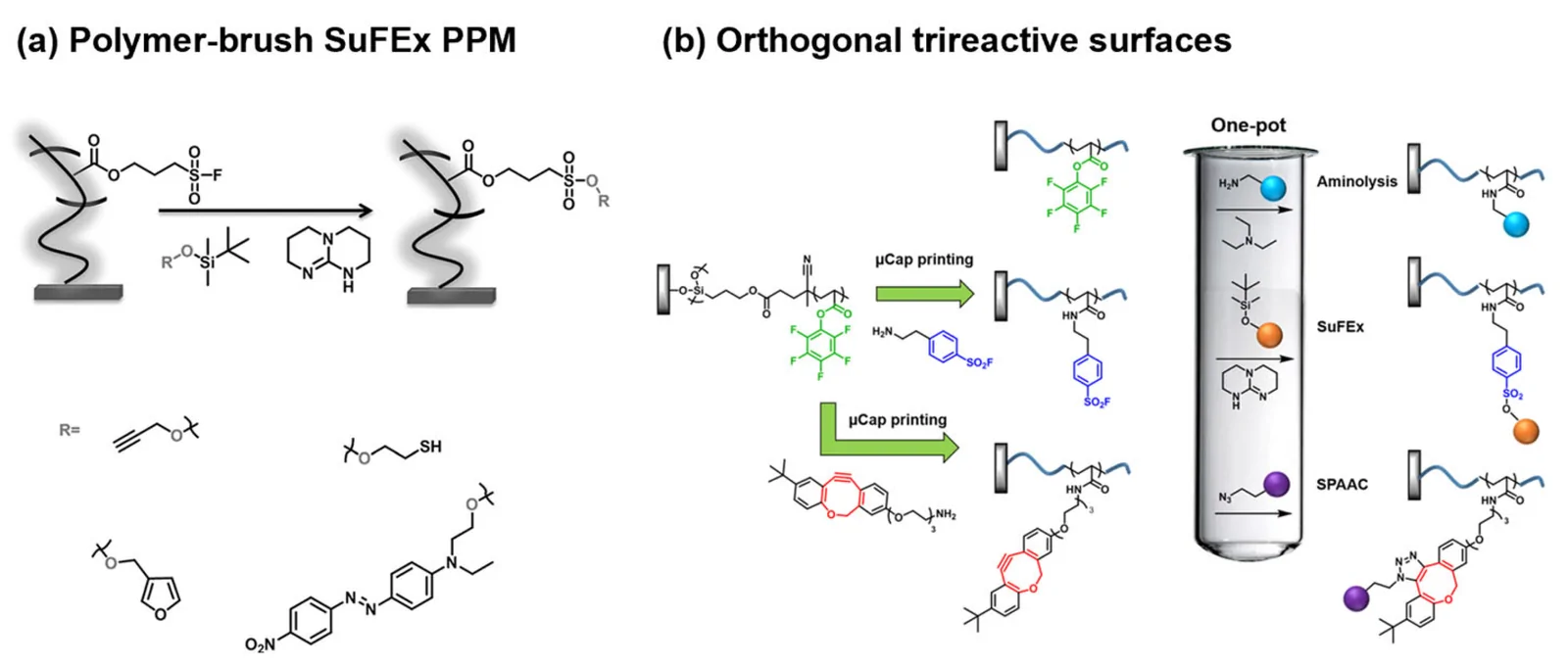
Surface-confined SuFEx platforms for polymer brushes and orthogonal interface patterning. (**a**) SuFEx post-functionalization of sulfonyl fluoride-bearing polymer brushes links a tethered chain ensemble to silyl ether partners under base catalysis. (**b**) Trireactive brush surfaces illustrate how aminolysis, SuFEx and SPAAC can be arranged as mutually addressable reaction channels for spatially differentiated interfaces.

Polymer brushes provide a well-defined platform for studying SuFEx under interfacial confinement. Surface-confined studies provide rare kinetic benchmarks for SuFEx under restricted mass transport. Sulfonyl fluoride-bearing polymer brushes showed rapid pseudo-first-order behavior, with reported apparent rate constants as high as 0.04 s^−1^ [34]. This approach exploited the high density and extended conformation of brush chains to generate functional surfaces that could not be accessed easily by direct polymerization of already functionalized monomers (Figure 9a). Subsequent kinetic studies compared alkyl sulfonyl fluorides, aryl sulfonyl fluorides and fluorosulfonates in brush environments and showed that apparent surface reactivity is governed by both the chemical identity of the S(VI)–F handle and the physical accessibility of the tethered layer [35]. Grafting density, brush swelling and catalyst penetration modulate the reaction in ways not fully captured by dilute-solution kinetics. The brush studies define a central theme for interfacial SuFEx: conversion is a coupled chemical and transport process. Dense brushes increase the number of potential reactive sites but may slow penetration of nucleophiles or catalysts; more open brushes improve access but reduce the maximum surface loading. Spectroscopic and wetting measurements provide useful average responses, yet they do not necessarily resolve depth-dependent conversion within the brush. The most informative surface studies therefore combine loss of the S(VI)–F signal, incorporation of the incoming group, retention of brush morphology and an application readout that is consistent with the installed functionality.

Orthogonal surface chemistry extends this logic from uniform derivatization to spatially programmed interfaces. Trireactive polymer brush platforms based on activated esters, strained alkynes and sulfonyl fluorides show that aminolysis, SPAAC and SuFEx can be conducted as mutually addressable reaction channels on the same surface (Figure 9b) [36]. Such systems illustrate the value of SuFEx as part of a broader interface-design toolkit: the reaction can be used not only to introduce one functional group, but also to preserve chemical information until a selected patterning or post-functionalization step is triggered.

Practical polymer-substrate coatings are less idealized than model brushes but demonstrate how SuFEx can be integrated into surface-engineering workflows. Tri-functional platforms combine SuFEx, photopolymerization and benzophenone photochemistry for one-pot dual-functional surfaces [37]. Related workflows adapt SuFEx to Y-shaped photoiniferter-modified PDMS [38] and benzophenone-assisted PVC surface functionalization [39]. These strategies are attractive because they adapt SuFEx to technologically relevant polymer substrates without requiring complete redesign of the bulk material. Their surface performance should be understood as the result of several coupled variables, including grafting density, coating thickness, roughness, residual low-molecular-weight species and the identity of the SuFEx-installed group.

Biological-interface coatings based on aliphatic polycarbonate brushes illustrate how similar SuFEx platforms can support distinct functional hypotheses. Ionic-liquid-functionalized brushes use late-stage SuFEx modification to introduce cationic antibacterial groups and combine this with self-polishing behavior [40]. PEG-functionalized analogs emphasize hydrophilic antifouling and dynamic antifouling, including enzyme-triggered surface renewal [41]. These examples show that SuFEx is most useful as a modular entry to families of related surfaces, rather than as a single determinant of biological performance. Antibacterial activity, fouling resistance and durability depend jointly on covalent composition, degradation rate, hydration, charge density and surface-regeneration behavior.

**Figure 10:**
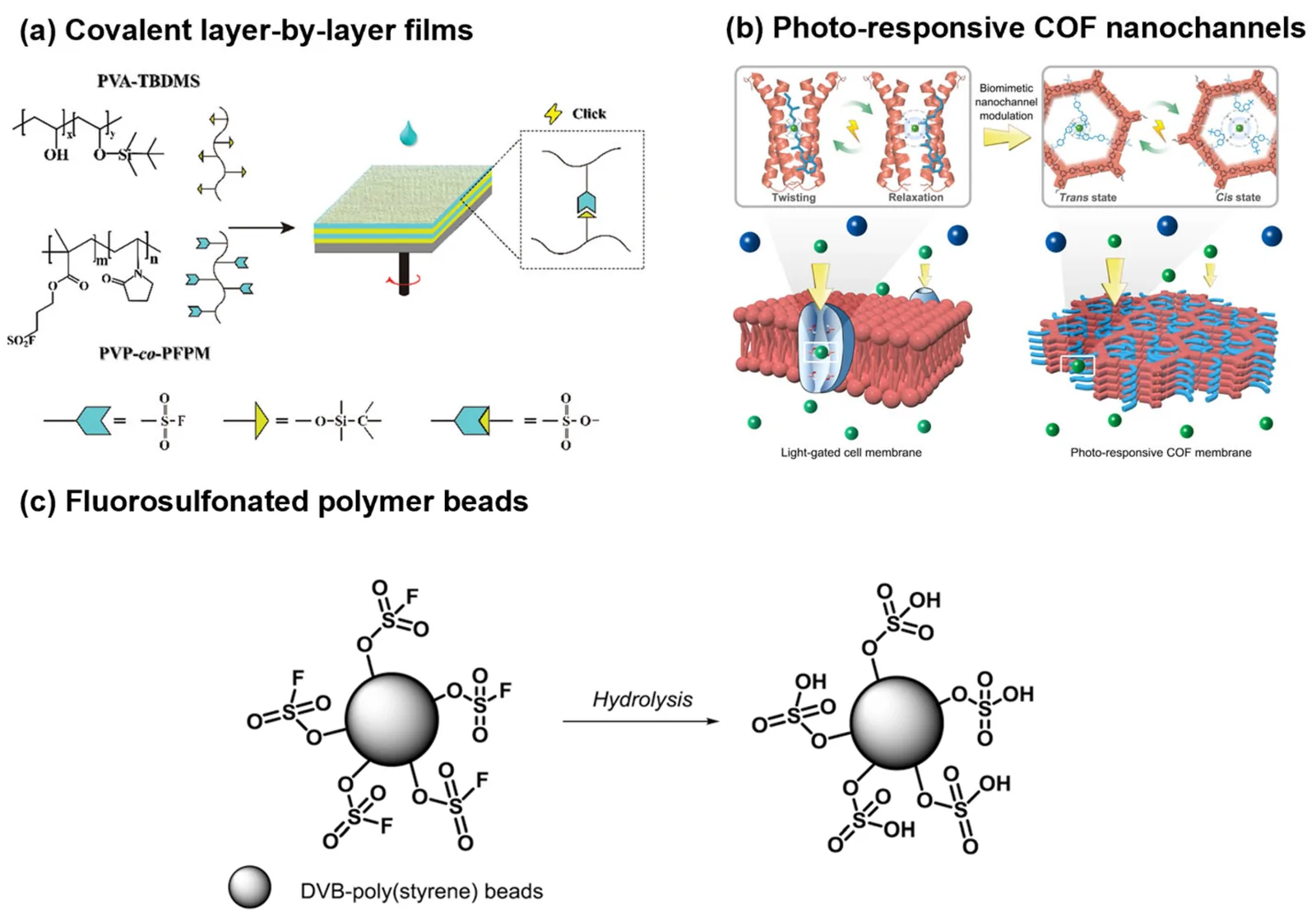
Confined polymer-derived architectures impose distinct accessibility tests on SuFEx chemistry. (**a**) Spin-assisted covalent layer-by-layer assembly uses complementary PVA-TBDMS/PVP-co-PFPM layers to generate reactive multilayer films. (**b**) Photo-responsive COF nanochannels translate SuFEx grafting into transport modulation within a porous membrane. (**c**) Fluorosulfonated polymer beads provide a high-curvature route to poly(hydrogen sulfate) particles and cation-exchange resins.

Covalent films and membranes introduce a further level of confinement because SuFEx can participate directly in film assembly or channel-wall modification. Reactive multilayer films prepared by spin-assisted layer-by-layer assembly of complementary PVA-TBDMS and PVP-co-PFPM use SuFEx to form covalent interlayer connections while retaining residual sulfonyl fluoride groups for subsequent functionalization (Figure 10a) [42]. In COF membranes, SuFEx grafting of internal nanochannel walls translates molecular modification into changes in pore size, charge distribution, hydration and ion transport (Figure 10b) [43]. These systems show the breadth of SuFEx-enabled membrane design, while also emphasizing that separation or transport performance arises from the interplay of covalent chemistry, morphology and confined mass transport.

Particles and cation-exchange resins occupy a related high-curvature regime. A mild SuFEx-based reagent system hydrolyzes fluorosulfonated polymer beads to poly(hydrogen sulfate) beads with predictable acid-site densities, which were then evaluated as cation-exchange resins in ion-capture experiments (Figure 10c) [44]. The same precursor chemistry also supports immobilization reactions with small molecules or proteins. Compared with planar brushes, particle systems offer higher accessible surface area and easier handling, but they also make spatial assignment more difficult because bulk composition, titration and ion-capture data average over outer surfaces and bead interiors.

Organic polymer surfaces and confined polymer-derived interfaces therefore reveal the principal promise of SuFEx at materials boundaries. The reaction can be embedded in brush PPM, coating fabrication, covalent film assembly, membrane nanochannel engineering and particle functionalization. As the geometry becomes more confined, the defining challenge shifts from demonstrating that SuFEx can occur to determining how uniformly it occurs and how durably the installed functionality persists under operating conditions. This perspective provides the link to carbon, inorganic and hybrid porous materials, where the surface no longer behaves as a swollen polymer layer but as a rigid or composite scaffold.

### 3.2. Carbon, Inorganic and Hybrid Porous Interfaces

Carbon, inorganic and hybrid porous interfaces expand SuFEx beyond organic polymer substrates while remaining directly relevant to polymer materials and composite design. These systems place different demands on the reaction. Carbon fibers require covalent surface modification that survives processing and improves fiber–matrix interactions. Model monolayers provide simplified platforms for quantifying surface-bound SuFEx reactivity. MOFs require post-synthetic modification without loss of crystallinity or porosity. Inorganic surfaces require stable anchoring and functional presentation across heterogeneous geometries. The common theme is the use of SuFEx to connect molecular functionality to a surface whose structure cannot be described by polymer molecular weight or dispersity.

**Figure 11:**
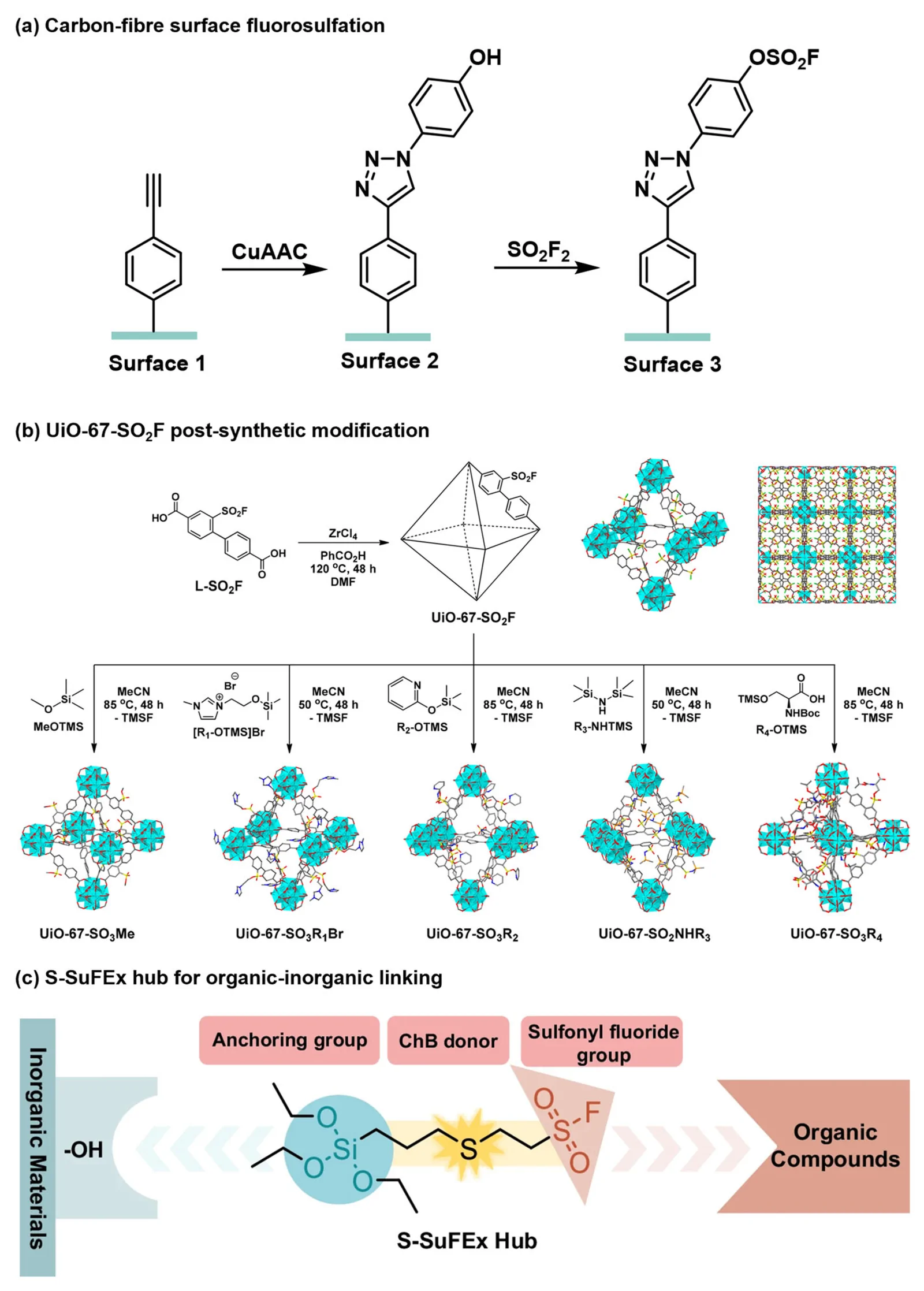
Carbon, MOF and inorganic interfaces illustrate the broadened evidence base for interfacial SuFEx. (**a**) Carbon-fiber modification uses pseudo-graft-from and pseudo-graft-to pathways to install fluorosulfate handles on non-polymeric composite-relevant surfaces. (**b**) UiO-67-SO2F post-synthetic modification shows how sulfonyl fluoride linkers can be exchanged while retaining a crystalline porous framework. (**c**) Chalcogen-bonding activation of alkyl sulfonyl fluorides enables S-SuFEx hubs for covalent organic–inorganic linking.

Carbon fiber modification represents a polymer-relevant boundary case because the substrate is non-polymeric, whereas the target function lies in composite interfaces. Installation of S(VI)–F functionality on carbon fibers followed by exchange with aryl silyl ethers enables pendant groups to be attached through sulfate linkages, and amino-functionalized fibers show improved interfacial shear strength (Figure 11a) [45]. This example broadens the role of SuFEx from polymer-chain modification to interface reinforcement. The relevant descriptors are correspondingly interfacial: surface composition, wetting, roughness, stability of the grafted layer, fiber morphology and mechanical coupling to the surrounding matrix.

Model monolayers offer complementary insight by removing much of the complexity associated with real composite or porous substrates. ESF-derived SuFExable monolayers enable quantitative surface conversion, di-SuFEx reactivity and dual CuAAC-SuFEx or SPOCQ-SuFEx sequences to be examined under controlled conditions [46]. These systems clarify how surface-bound S(VI)–F groups can be installed, exchanged and combined with other click reactions. Although monolayers do not reproduce the swelling, roughness or mechanical stresses of practical interfaces, they provide a chemically precise benchmark for interpreting more complex SuFEx-modified surfaces.

MOFs illustrate the additional requirements imposed by porous crystalline scaffolds. Sulfonyl fluoride-containing UiO-67 frameworks can undergo SuFEx post-synthetic modification to introduce new functionality while maintaining the MOF structure (Figure 11b) [47]. The significance of this work lies in coupling covalent exchange to framework retention: a successful modification must preserve crystallinity, porosity and accessibility rather than simply demonstrate reaction of a linker. Imidazolium-functionalized UiO-67 materials used for heterogeneous catalysis show how SuFEx can convert a stable porous scaffold into a functional solid, provided that the catalytic behavior is interpreted together with retained framework structure and accessible internal sites.

Recent organic–inorganic linking based on intramolecular chalcogen-bond activation further expands the range of SuFEx hubs available for hybrid interfaces (Figure 11c) [48]. Trialkoxysilane-bearing alkyl sulfonyl fluorides combine inorganic surface anchoring with activated S(VI)–F exchange, enabling antibacterial, hydrophobic and fluorescent functions to be presented on inorganic substrates. This chemistry is important because it addresses a long-standing limitation of alkyl sulfonyl fluorides, whose lower reactivity has restricted their use in SuFEx with alcohols and amines. The resulting S-SuFEx design points toward more general organic–inorganic coupling strategies in which the activating element, anchoring group and functional payload are integrated into a single molecular platform.

Taken together, these studies show that interfacial SuFEx is best viewed as a post-formation covalent strategy for functionalizing preassembled material architectures. Its value lies not simply in transferring solution-phase PPM to a surface, but in enabling S(VI)–F exchange within environments where tethering, swelling, curvature, pore confinement and framework rigidity determine whether reactive sites remain accessible. In polymer brushes and coatings, this logic supports late-stage functional installation in tethered or surface-bound layers; in multilayer films, COF nanochannels and particles, it links covalent assembly or pore-wall modification to transport and ion-exchange functions; and in carbon, MOF and inorganic systems, it provides polymer-relevant routes to composite adhesion, porous-framework post-synthetic modification and hybrid organic–inorganic coupling. Future progress will depend less on demonstrating that surface SuFEx can occur than on controlling where it occurs, how completely it proceeds, how stable the modified interface remains, and how directly the installed functionality accounts for the measured performance. This emphasis on accessibility, scaffold preservation and structure–function assignment provides a natural transition to biopolymer systems, where the same interfacial constraints are further complicated by sequence, recognition, folding and competing biological nucleophiles.

## 4. SuFEx Modification of Natural Polymers and Biopolymers

Natural polymers and sequence-defined biopolymers introduce a distinct set of constraints for SuFEx chemistry. Unlike synthetic polymer scaffolds, whose reactive sites are often installed by monomer design or post-polymerization chemistry, biopolymers present preorganized arrays of hydroxyl, amino, carboxylate, phosphate and aromatic residues within hydrated, folded, crystalline, supramolecular or recognition-defined environments. In these systems, the apparent selectivity of an S(VI)–F handle is rarely a purely intrinsic property of the warhead. It emerges from accessibility, local concentration, binding geometry, scaffold dynamics and competition with other nucleophiles. SuFEx modification of biopolymers is therefore best discussed along two connected axes: materials-level functionalization of natural polymer scaffolds and recognition-guided covalent capture in sequence-defined biomolecules.

This section focuses on cases in which SuFEx contributes to post-formation modification, conjugation, cross-linking, covalent capture or functional assembly of a natural polymer, peptide, protein, nucleic acid or aptamer system. The discussion does not attempt to cover all SuFEx chemical-biology probes or covalent drug designs. Instead, emphasis is placed on how the covalent step is integrated with a macromolecular or biomolecular scaffold. The key distinction from synthetic polymer PPM is that function often depends as much on scaffold organization and molecular recognition as on the new S(VI)-centered linkage itself.

### 4.1. Polysaccharide-Based Natural Polymers and Glycan-Rich Interfaces

Polysaccharide-based systems place SuFEx in hydroxyl- and amino-rich matrices whose reactivity is shaped by crystallinity, fiber morphology, hydration and processing. In this class, SuFEx has been used in three main ways: to functionalize composite polysaccharide surfaces, to construct or reinforce processed cellulose networks and to prepare chitosan conjugates that subsequently assemble into hydrogels. Glycan-rich biological interfaces form a related but mechanistically distinct category, because covalent capture is driven by recognition between a protein scaffold and a carbohydrate target rather than by bulk polysaccharide modification.

**Figure 12:**
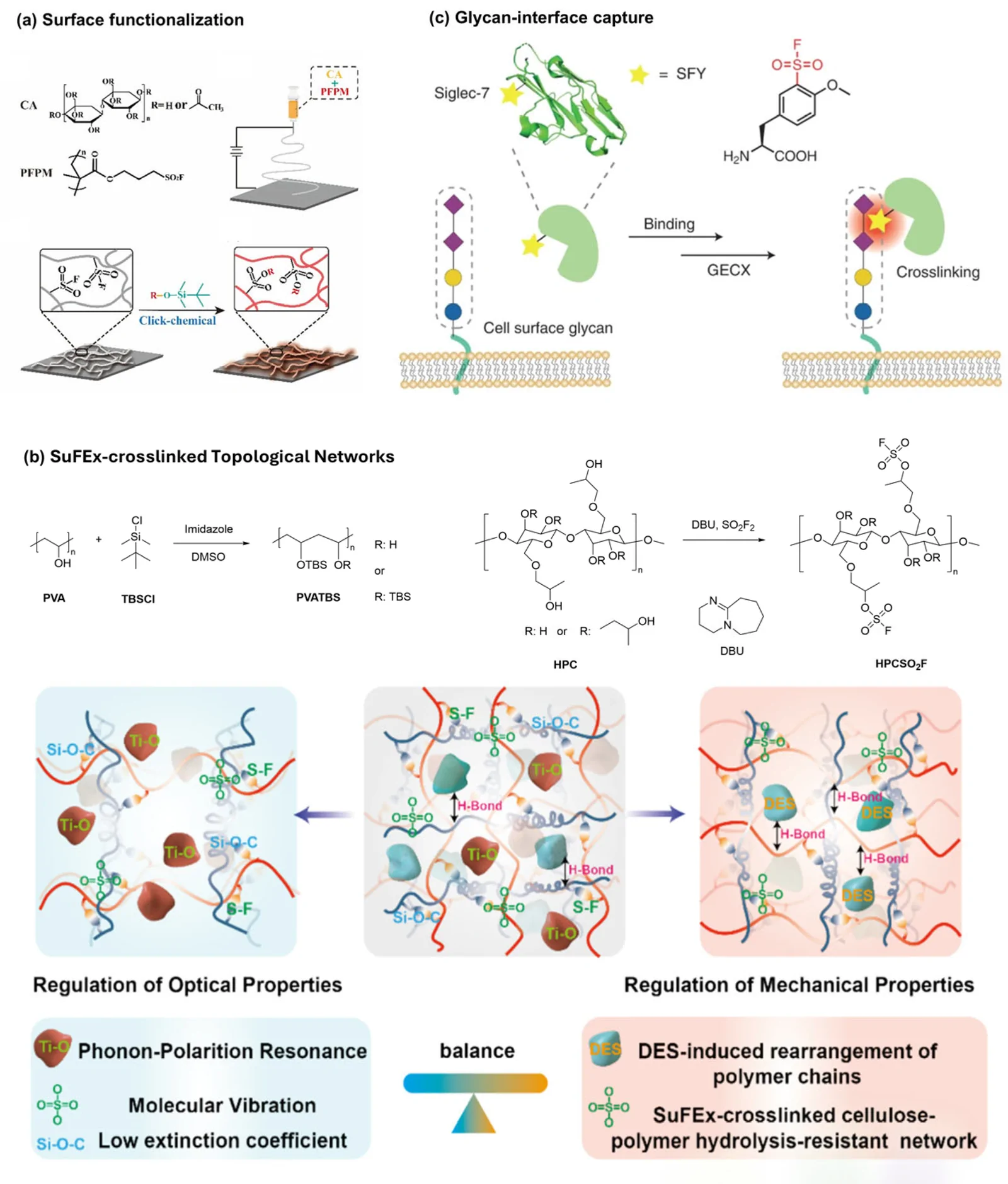
SuFEx chemistry in polysaccharide-based materials and glycan-rich biological interfaces. (**a**) SuFEx-functionalizable CA/PFPM nanofibers for modular surface modification. (**b**) SuFEx-linked cellulose/PVA networks for thermal metamaterials. (**c**) Genetically encoded S(VI)–F chemistry enables proximity-driven covalent capture of sialoglycans.

Electrospun cellulose acetate/poly(3-(fluorosulfonyl)propyl methacrylate) composite nanofibers bearing sulfonyl fluoride sites provide an early example of SuFEx-enabled polysaccharide-derived surface functionalization (Figure 12a) [49]. The SO_2_F-containing composite fibers can be modified with silyl ether dyes, PEG chains, ionic-liquid derivatives and secondary click handles, enabling antifouling or antibacterial surface properties. This work is best viewed as SuFEx functionalization of an engineered composite nanofibre rather than direct derivatization of neat cellulose acetate. Its broader significance lies in showing how a polysaccharide-containing material can be converted into a modular biofunctional surface when reactive handles, fiber morphology and surface presentation are designed together.

Processed cellulose and chitosan systems illustrate the transition from surface derivatization to functional material formation. In cellulose thermal metamaterials, a silyl-protected PVA component and an SO_2_F-bearing cellulose derivative form a SuFEx-linked network, while deep eutectic solvent treatment, UV polymerization and TiO_2_ scattering contribute to chain confinement, mechanical resilience and passive radiative cooling (Figure 12b) [50]. SuFEx is therefore one element of an integrated materials design rather than the sole origin of thermal performance. Chitosan–PEG conjugates represent a different mode of operation: sulfonyl fluoride functionality is installed on chitosan and coupled with mPEG-NH_2_ through SuFEx, after which Pluronic F127/alpha-cyclodextrin host–guest interactions generate supramolecular hydrogels for protein loading and release [51].

Glycan-rich interfaces extend SuFEx into proximity-enabled carbohydrate capture. Genetically encoded sulfonyl fluoride-containing amino acids have been used to form covalent linkages between proteins and carbohydrates under biocompatible conditions (Figure 12c) [52]. This chemistry should not be classified as bulk polysaccharide functionalization. Its importance lies in converting weak protein–carbohydrate recognition into a covalent readout when the S(VI)–F handle is placed close to a suitable glycan nucleophile. Thus, polysaccharide and glycan-related SuFEx spans a broad continuum, from materials surface modification to recognition-controlled covalent locking at biological interfaces.

### 4.2. Peptide-, Polypeptide- and Protein-Based Biopolymers

Peptide, polypeptide and protein systems provide the most diverse biopolymer landscape for SuFEx. They range from proteinaceous materials and synthetic polypeptides to folded-protein conjugation, genetically encoded covalent capture, cross-linking mass spectrometry, reactive therapeutics and peptide macrocyclization. A useful organizing principle is scaffold hierarchy. Material-scale systems use SuFEx to alter bulk or interfacial properties of amino-acid-derived polymers; folded proteins require preservation of structure and site assignment; genetically encoded systems use proximity to create selectivity; and peptides use intramolecular or affinity-guided geometry to control topology or capture.

**Figure 13:**
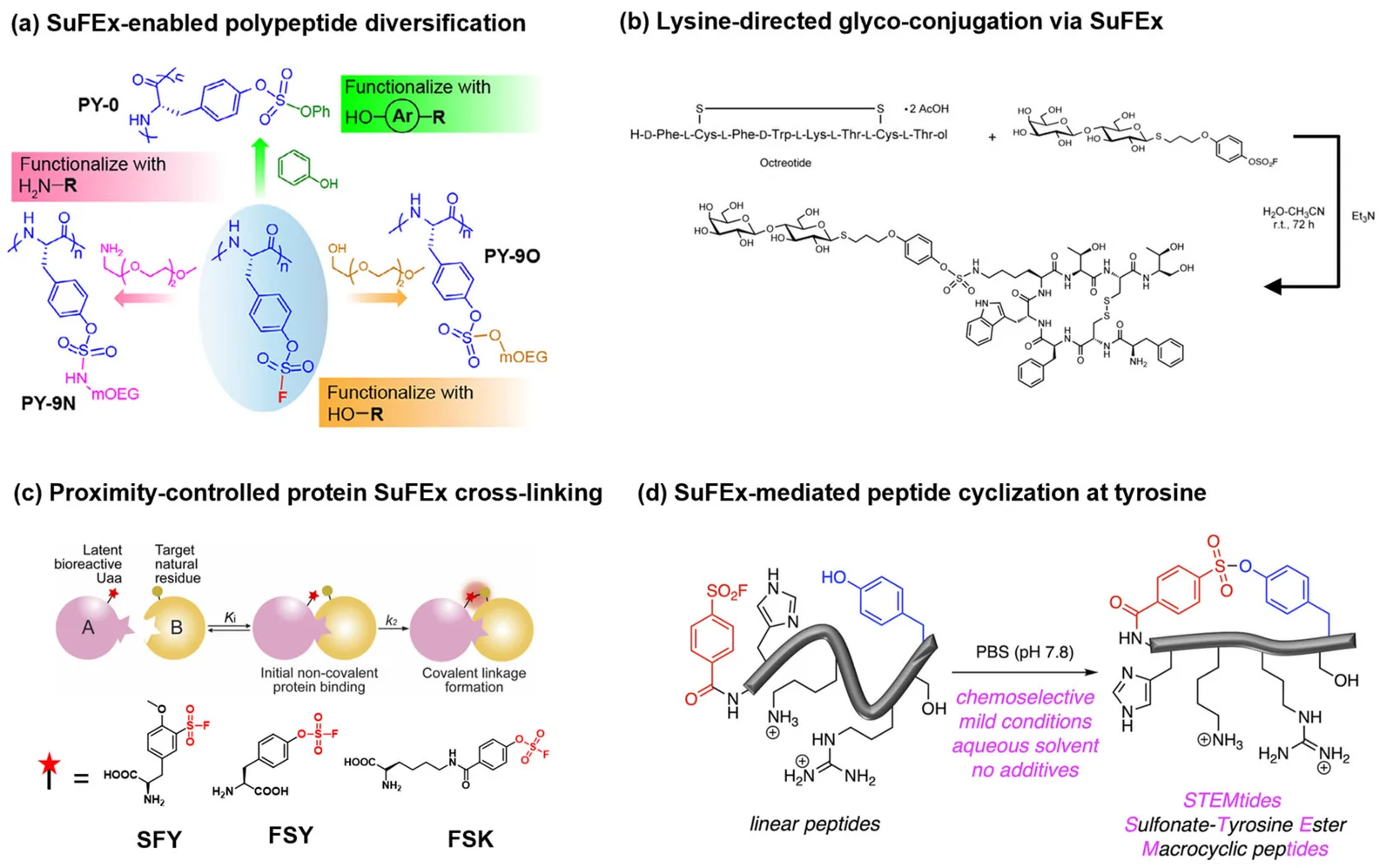
Scaffold-dependent SuFEx modes in peptide-, polypeptide- and protein-based biopolymers. (**a**) Side-chain S(VI)–F-containing polypeptides serve as material-scale scaffolds for SuFEx diversification. (**b**) Fluorosulfate thiolactosides enable lysine-directed glyco-conjugation of peptides and folded proteins. (**c**) FSY, FSK and SFY illustrate genetically encoded SuFEx handles for proximity-controlled protein cross-linking. (**d**) Peptide-level SuFEx enables topology control through tyrosine-selective intramolecular macrocyclization.

At the materials scale, silk fibroin aerogels and synthetic polypeptides show how SuFEx can tune amino-acid-containing polymer scaffolds. SuFEx modification of tyrosine residues in silk fibroin silicon aerogels introduces quaternary-ammonium functionality before aerogel formation, producing materials with anion–dye adsorption and antibacterial performance relevant to water-treatment concepts [53]. The resulting performance reflects both covalent installation and aerogel architecture, including charge density, porosity, silicon-network formation and water-accessible morphology. SuFEx-enabled polypeptides prepared from O-fluorosulfonyl tyrosine-derived NCA monomers provide a more sequence-inspired platform (Figure 13a) [54]. Ring-opening polymerization generates side-chain S(VI)–F-containing polypeptides that can be diversified with both small molecules (alcohols, phenols, and amines) and saccharide-containing macromolecules to form sulfate- or sulfamate-linked products. In this case, the linkage itself becomes a design variable that influences solubility, conformation, morphology and cell interactions.

Folded-protein modification shifts the emphasis from polymer architecture to site distribution and structural integrity. Early work on direct introduction of R–SO_2_F motifs into proteins and protein–polymer conjugation demonstrated that SuFEx chemistry can be applied to biomacromolecules such as BSA [55]. Gel electrophoresis, MALDI-TOF MS and FTIR supported covalent modification, but these ensemble methods do not fully resolve modification sites, product heterogeneity or higher-order structure. Fluorosulfate thiolactosides provide a more residue-directed example by installing lactose motifs on lysine-containing proteins, including ubiquitin and L-asparaginase II, through sulfamoyl-type linkages (Figure 13b) [56]. These studies show that SuFEx can contribute to protein glycoengineering, while also underscoring the need to distinguish in vitro stabilization from broader pharmacokinetic or therapeutic claims.

Genetic-code expansion transforms SuFEx from available-residue modification into proximity-controlled covalent chemistry. Fluorosulfate-L-tyrosine introduces a latent aryl fluorosulfate warhead that reacts with nearby Lys, His or Tyr residues only when folding or binding places a suitable nucleophile within reach [57]. Fluorosulfonyloxybenzoyl-L-lysine extends the reaction radius through a longer side chain and expands the range of accessible protein contexts [58]. Mechanistic studies further establish that protein SuFEx is a two-step process in which noncovalent association precedes covalent bond formation (Figure 13c) [59]. Binding affinity, warhead placement, target-residue identity, pH, local structure and competing nucleophiles together determine the final cross-linking outcome. This behavior distinguishes protein-context SuFEx from globally selective bioconjugation and aligns it more closely with recognition-guided covalent capture.

The same proximity principle underlies SuFEx applications in structural proteomics and reactive protein therapeutics. The NHSF cross-linker uses an NHS ester to install the reagent on lysine and an aryl sulfonyl fluoride to capture proximal residues, expanding the set of distance restraints available for cross-linking mass spectrometry [60]. Its value is analytical rather than materials-oriented: SuFEx converts spatial proximity into a covalent mass-spectrometric constraint. Proximity-enabled reactive therapeutics, exemplified by FSY-containing PD-1 variants that covalently engage PD-L1, show how genetically encoded SuFEx can enhance biological activity through target locking [61]. In these cases, covalent capture must be interpreted together with protein expression, folding, target binding, delivery, pharmacology and off-target reactivity.

Recent peptide macrocyclization provides an example in which reaction conditions, isolated yield, and chemoselectivity were reported together. A tyrosine-containing model peptide underwent full conversion in PBS at pH 7.8 and ambient temperature within 2 h, affording the macrocycle in 95% isolated yield (Figure 13d) [62]. Entry-inhibitor peptides bearing a PEG spacer and sulfonyl fluoride warhead instead use target recognition to covalently capture SARS-CoV-2 RBD variants and enrich pseudovirus from wastewater matrices on magnetic beads [63]. The first case uses SuFEx to define intramolecular architecture, whereas the second uses it to lock a recognition complex. Together, these examples show that peptide SuFEx is most powerful when the reactive handle is positioned by sequence, conformation or target binding rather than by indiscriminate electrophilicity.

Across peptide and protein systems, SuFEx functions as a scaffold-dependent covalent strategy rather than a universally selective warhead. Its strongest uses combine latent reactivity with molecular organization: polypeptide side chains tune material properties, folded proteins direct residue-level modification, genetically encoded handles exploit proximity, and peptides use intramolecular or target-guided geometry. The same features also define the main limitations, because functional outcomes can arise from altered charge, folding perturbation, affinity, supramolecular assembly or nonspecific labeling as well as from the intended covalent linkage.

### 4.3. Nucleic Acid and Aptamer-Based Biopolymers

Nucleic acid and aptamer systems extend SuFEx into sequence-programmed recognition. In these systems, the nucleic acid often supplies binding information, whereas the S(VI)–F handle supplies irreversible engagement once a suitable nucleophile is positioned nearby. The central design problem is therefore the balance between affinity and reactivity: strong binding alone does not ensure covalent capture, and a highly reactive warhead can increase nonspecific labeling. This section treats nucleic-acid SuFEx as recognition-guided covalent locking rather than as ordinary nucleic-acid functionalization.

**Figure 14:**
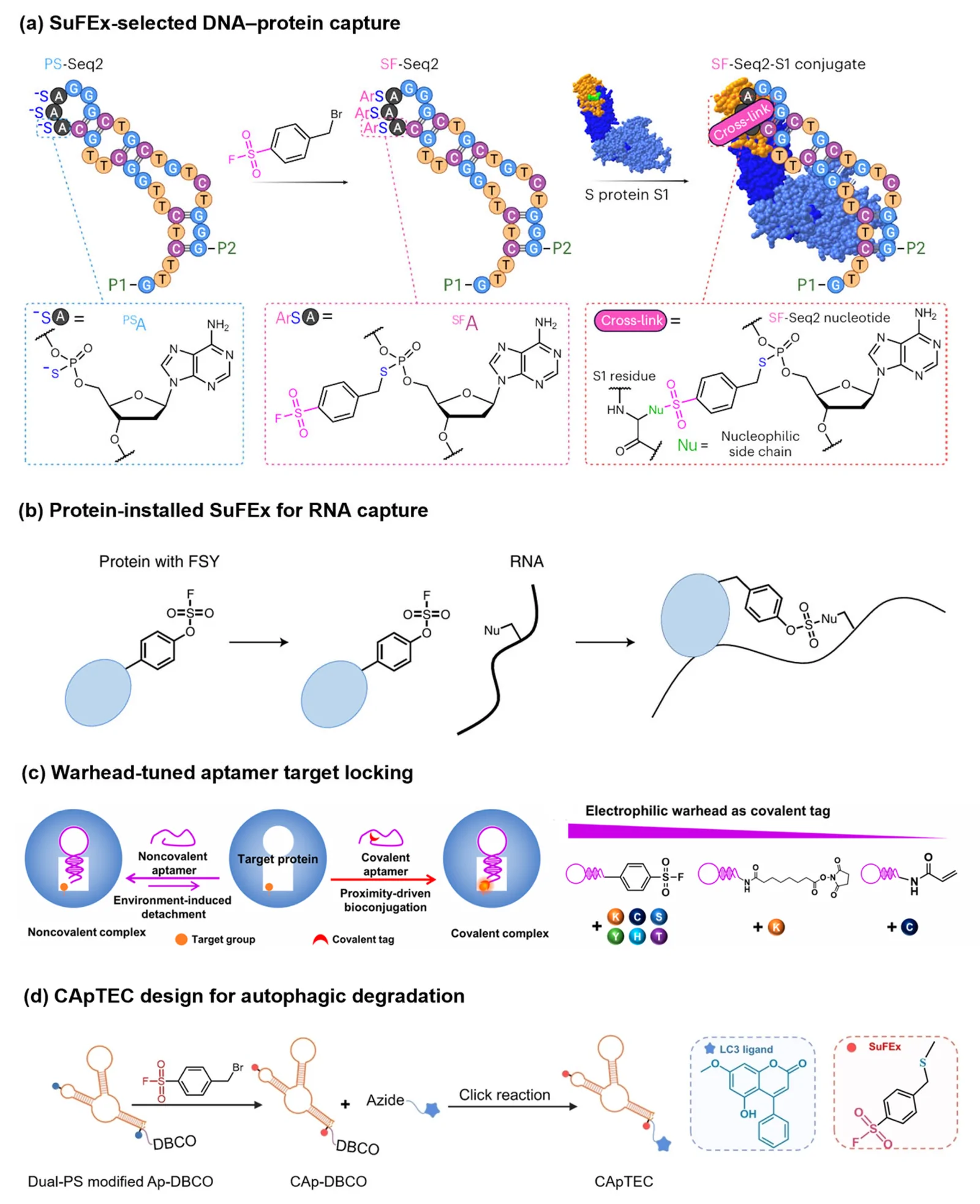
Recognition-guided SuFEx strategies in nucleic acid and aptamer biopolymers. (**a**) SuFEx-selected DNA ligands position aryl sulfonyl fluoride groups near nucleophilic residues on S1, enabling covalent DNA–protein capture. (**b**) In genetically encoded RNA cross-linking, the SuFEx-capable amino acid is installed on the RNA-binding protein to capture bound RNA through proximity-driven covalent reaction. (**c**) Covalent aptamers convert reversible aptamer–protein binding into persistent target locking. (**d**) CApTEC combines aptamer recognition, sulfonyl fluoride-mediated covalent locking and LC3 recruitment to promote autophagic degradation of membrane proteins.

SuFEx-modified oligonucleotide libraries provide a clear example of this affinity–reactivity coupling. Aryl sulfonyl fluoride or aryl fluorosulfate warheads can be installed onto phosphorothioate-containing DNA after PCR through phosphorothioate-bromide chemistry, enabling very large libraries to be selected for covalent reaction with protein targets (Figure 14a) [64]. The platform is powerful because it can identify ligands that both bind and position a warhead near a reactive residue at a protein–protein interface. Genetically encoded protein–RNA cross-linking offers a related but mechanistically different case (Figure 14b) [65]. There, the SuFEx-capable amino acid is installed on the protein rather than on RNA, so the reaction reports protein–RNA proximity instead of directly functionalizing the nucleic acid scaffold.

Covalent aptamers illustrate the benefit–risk balance of irreversible target locking. Comparative SARS-CoV-2 covalent-aptamer studies show that while sulfonyl fluoride-modified aptamers can highly efficiently cross-link target proteins, their increased reactivity also enhances unwanted nonspecific labeling in protein-rich media when compared to NHS ester and acrylamide warheads (Figure 14c) [66]. Covalent aptamer chimeras further integrate recognition, SuFEx locking and induced degradation. Site-specific sulfonyl fluoride installation on aptamers, combined with an LC3 ligand, enables covalent aptamer-based autophagosome-tethering chimeras for membrane protein degradation (Figure 14d) [67].

Nucleic acid and aptamer SuFEx therefore occupy the interface between chemical biology and polymer conjugation. The nucleic acid provides a programmable recognition scaffold, while SuFEx introduces covalent persistence. The most informative studies separate reversible affinity, warhead placement, target-site engagement, nonspecific labeling and downstream function. This distinction is essential because cross-linking, inhibition, enrichment and degradation are related but not equivalent outcomes.

Taken together, biopolymer SuFEx is most compelling when covalent exchange is organized by scaffold architecture or molecular recognition. Polysaccharide-derived materials demonstrate surface functionalization, network formation and precursor assembly; peptide and protein systems illustrate material tuning, residue-level conjugation, proximity-enabled cross-linking and topology control; nucleic acid and aptamer systems convert sequence recognition into persistent capture or degradation. The future value of SuFEx in biopolymer functionalization will depend on maintaining this connection between chemical evidence, site or interface assignment, scaffold integrity and function-specific controls.

## 5. Outlook and Conclusions

SuFEx chemistry has emerged as a sulfur(VI)-centered click platform defined by the unusual stability–reactivity balance of S(VI)–F bonds. Sulfonyl fluorides, fluorosulfates, sulfamoyl fluorides and related SuFExable hubs can tolerate many synthetic, processing and biological conditions, yet undergo exchange with suitable nucleophilic partners under appropriate activation or local recognition. This programmable latency distinguishes SuFEx from many conventional coupling reactions. The S(VI)–F group is not simply a reactive functional handle, but a tunable connector whose behavior is governed by hub structure, nucleophile identity, activation mode and reaction environment.

This reactivity profile is particularly valuable for polymer functionalization because it allows scaffold construction and functional installation to be separated in time and space. Either the polymer scaffold or the incoming modifier can carry the S(VI)–F handle, enabling post-polymerization side-chain modification, chain-end transformation, macromolecular coupling, surface derivatization, pore-wall modification and biopolymer conjugation. Across these settings, SuFEx has demonstrated the ability to diversify common polymer precursors, functionalize preformed interfaces, operate within confined architectures and convert molecular recognition into covalent capture.

Future progress will depend on two connected advances. At the reaction level, more predictive hub design, milder activation modes, aqueous-compatible protocols and clearer kinetic understanding will broaden the usable SuFEx toolbox.

In addition, recent studies demonstrated the emerging potential of artificial intelligence for SuFEx design at both the reaction and polymer-material levels, providing a methodological basis for extending data-driven workflows to SuFEx-mediated polymer functionalization, including the prioritization of reaction partners and conditions according to conversion, scaffold integrity, and target material properties [68,69]. The increasing availability of aqueous and biocompatible SuFEx protocols points toward greener polymer functionalization, yet systematic studies on solvent recyclability, catalyst recovery, and life-cycle assessment for SuFEx-modified materials remain to be addressed [70,71,72,73]. From a process-engineering perspective, recent flow-based SO_2_F_2_ platforms provide useful precedents for continuous and scalable SuFEx implementation, while broader translation will require systematic assessment of process economics, gas-handling safety, and application-specific regulatory requirements [74,75].

At the application level, SuFEx is well positioned for late-stage modification of degradable, renewable and commodity polymers, orthogonal surface patterning, membrane and film engineering, porous and hybrid materials, peptide macrocyclization, programmable bioconjugation and covalent aptamer or oligonucleotide systems. Coupled with rigorous evidence for conversion, scaffold integrity and structure–function relationships, SuFEx chemistry should continue to develop from a powerful molecular ligation strategy into a general platform for precision polymer modification, interface engineering and recognition-guided biopolymer conjugation.

## Data Availability

No new data were created or analyzed in this study. Data sharing is not applicable to this article.

## Abbreviations

The following abbreviations are used in this manuscript:

AGET	activators generated by electron transfer
ATRP	atom transfer radical polymerization
AEM	anion-exchange membrane
CA	cellulose acetate
COF	covalent organic framework
CuAAC	copper-catalyzed azide-alkyne cycloaddition
DART-HRMS	direct analysis in real time-high-resolution mass spectrometry
DBU	1,8-diazabicyclo [5.4.0]undec-7-ene
FSY	fluorosulfate-L-tyrosine
HAT	hydrogen-atom transfer
LC3	microtubule-associated protein 1A/1B-light chain 3
MOF	metal–organic framework
NCA	N-carboxyanhydride
NHSF	N-hydroxysulfosuccinimide and aryl sulfonyl fluoride moieties
PDMS	polydimethylsiloxane
PEI/bPEI	polyethylenimine/branched polyethylenimine
PPM	post-polymerization modification
RAFT	reversible addition-fragmentation chain-transfer polymerization
RBD	receptor-binding domain
ROMP	ring-opening metathesis polymerization
SEC/GPC	size-exclusion chromatography/gel permeation chromatography
SPAAC	strain-promoted azide-alkyne cycloaddition
SPOCQ	strain-promoted oxidation-controlled cyclooctyne-quinone cycloaddition
SuFEx	sulfur(VI) fluoride exchange
TBD	1,5,7-triazabicyclo [4.4.0]dec-5-ene
TBDMS	tert-butyldimethylsilyl
TMS	trimethylsilyl
XPS	X-ray photoelectron spectroscopy
